# Maintenance and Neuronal Differentiation of Chicken Induced Pluripotent Stem-Like Cells

**DOI:** 10.1155/2014/182737

**Published:** 2014-12-09

**Authors:** Rui Dai, Ricardo Rossello, Chun-chun Chen, Joeran Kessler, Ian Davison, Ute Hochgeschwender, Erich D. Jarvis

**Affiliations:** ^1^Department of Neurobiology, Duke University Medical Center and Howard Hughes Medical Institute, P.O. Box 3209, Durham, NC 27710, USA; ^2^Instituto de Células Madres SUAGM, Sistema Universitario Ana G. Méndez, P.O. Box 21345, San Juan, PR 00928-1345, USA; ^3^Max-Delbrück Center for Molecular Medicine, Berlin Brandenburg School for Regenerative Therapies, Hochbau 31.1, 13125 Berlin, Germany; ^4^Department of Biology, Boston University, LSEB 406, Boston, MA 02215, USA; ^5^Neurotransgenic Laboratory, Duke University Medical Center, P.O. Box 3209, Durham, NC 27710, USA

## Abstract

Pluripotent stem cells have the potential to become any cell in the adult body, including neurons and glia. Avian stem cells could be used to study questions, like vocal learning, that would be difficult to examine with traditional mouse models. Induced pluripotent stem cells (iPSCs) are differentiated cells that have been reprogrammed to a pluripotent stem cell state, usually using inducing genes or other molecules. We recently succeeded in generating avian iPSC-like cells using mammalian genes, overcoming a limitation in the generation and use of iPSCs in nonmammalian species (Rosselló et al., 2013). However, there were no established optimal cell culture conditions for avian iPSCs to establish long-term cell lines and thus to study neuronal differentiation *in vitro*. Here we present an efficient method of maintaining chicken iPSC-like cells and for differentiating them into action potential generating neurons.

## 1. Introduction

Pluripotent stem cells are undifferentiated cells that have the potential to become any cell type in the adult body. They have two main hallmarks. First, unlike differentiated somatic cells, stem cells have the ability to self-renew and proliferate. Second, they have the ability to differentiate into somatic cells with drastically different properties. There are currently two main methods that can be used to derive or create stem cells* in vitro*. The first is to isolate embryonic stem cells (ESC) from early embryos. The second is to induce pluripotent stem cells (iPSCs) from somatic cells such as fibroblasts, first demonstrated possible with a combination of four transcription factors: Oct4, Sox2, Klf4, and c-myc [1].

Though both ESCs and iPSCs can differentiate into somatic cells, there are differences between the two types of stem cells that still elicit concerns of whether iPSCs could fully recapitulate the characteristics of pluripotent ES cells. These include iPSCs genetic alterations from integration of the viral vector containing the reprogramming transcription factors into host genes [2] and epigenetic modifications such as DNA methylation and histone activation [3]. Even after reprogramming, iPSCs still retain memory of the tissue that they were derived from (although possibly limited to early passages), which might restrict their differentiation into other types of cells [4, 5]. Sometimes the differentiation restriction decreases as the number of passages of iPSCs increases [6]. Thus, because of possible differences between ESCs and iPSCs, it is important to verify the differential and developmental properties of any line of iPSCs generated.

Birds are important organisms for studying development, including neural development [7]. One of the advantages to using chicken as a model organism is the ability to monitor its embryo development* in ovo*. However, a limitation of studies with birds is that there are very few cell lines available for studying cell function. Thus, one of our goals has been to create avian stem cells that could be used to study neuronal function. Towards this goal, we recently were successful at using the mammalian homologs of the iPSC inducing transcription factors to induce partial iPSC-like cells across the animal kingdom, in birds, fish, and* Drosophila* [8]. We were able to show* in vivo* incorporation and differentiation of the cells in birds and fish but were not able to obtain stable cell lines that survived many passages. Here we developed novel media conditions that allowed us to maintain these same avian cells well past 20 passages, and we were able to differentiate them into neurons* in vitro*.

## 2. Materials and Methods

The starting fibroblasts and initial iPSC-like cells were the same as the cells in Rosselló et al. [8]. However, unlike in that study, in this study after the first week of transduction we compared the growth of the cells in different media conditions. In the previous study, where relevant, a citation was mentioned to the current study as unpublished findings in preparation.

### 2.1. Embryonic Fibroblast Extraction

As described in Rosselló et al. [8], to obtain embryonic fibroblasts, fresh chicken eggs were incubated at 37°C for 2–4 days and mouse embryos were collected at embryonic day 12. Embryos were extracted and the abdomen and head separated with forceps. The rest of the embryo was dissociated and trypsinized with 0.25% trypsin with EDTA (Gibco) at 37°C until single cell suspension was obtained. The trypsin was then neutralized with KO DMEM (Gibco 10829) plus 15% FBS (HyClone). The cells were spun down for 5–7 minutes at 1500 rpm and the supernatant was aspirated. The cells were then plated in 15 cm cell culture plates at 1 embryo per plate with KO DMEM plus 15% FBS and incubated at 39°C (chicken) or 37°C (mouse), respectively, in a 5% CO_2_ atmosphere with 21% oxygen. After two days of incubation, the cells were passaged once at a 1 : 4 dilution and frozen or transfected two days later as described below.

### 2.2. Induced Pluripotent Stem Cell Transduction and Culture

As described in Rosselló et al. [8], chicken or mouse embryonic fibroblasts were transduced in 2i+ modified medium (see below) with the STEMCCA viral vector containing the mouse Oct4, Sox2, Klf4, and c-myc genes according to the ViraDuctin Lentivirus Transduction Kit (LTV-201) [9]. We typically transduced 12–24 independent fibroblast replicate cultures in 12-well cell culture plates (Corning, 3.8 cm^2^). A week after transduction in cESC media, the cells were then passaged in many different media condition combinations by trial and error in 4 independent replicates for each condition, of which five we report here for comparative purposes (Table 1 and see below). Since different media conditions were conducted at the same time in 4 wells each of 12–24 well plates, this controlled for experimental plate, incubator, or other variables.

### 2.3. Growth Media Conditions

*BRL (Buffalo Rat Liver) Conditioned Media Plus*. 90% KO DMEM (Sigma) + 10% FBS (Hyclone) was preconditioned with Buffalo Rat Liver cells for 3 days to create BRL conditioned media. Then for every 100 mL of BRL conditioned media, we added the following standard cell culture ingredients (generating 150 mL total): KO DMEM (15 mL), FBS (7.5 mL), 100 mM sodium pyruvate (1.5 mL), 100x nonessential amino acids (1.5 mL; Sigma), 100x Penicillin-Streptomycin (1.5 mL, Sigma), 100x nucleosides (1.5 mL, Millipore), 100x Glutamax (250 *μ*L, Life Technology), and beta-mercaptoethanol (190 *μ*L, Sigma) to generate “BRL conditioned plus” media, with the final concentrations as listed in Table 1. This medium has been used to grow chicken primordial germ cells (PGCs; [10]), except that we omitted the chicken serum.

*cESC (Chicken Embryonic Stem Cell) Media*. This medium is based on Samarut and Pain [11] which was designed to support proliferation of chicken ESCs. It was used to grow our initial iPSC-like avian cells that lasted up to the 5th passage reported in Rosselló et al. [8]. It consists of standard media with differences in FBS and Glutamax concentrations and with LIF (Millipore LIF2010) and the following recombinant cytokines added (Table 1): rhIL6 (Peprotech 200-06), rhsIL6Ra (Peprotech 200-06R), rmSCF (Peprotech 250-03), rhIGF1 (Peprotech 100-11), and rhFGF2 (Peprotech 100-18B). The purposes of the LIF and recombinant cytokines from human (h) and mouse (m) are to inhibit differentiation.

*2i+ (Three Inhibitor) *+* LIF Media (2i+)*. The 2i+ medium was based on [12–14] and contains 3 chemicals that inhibit cell differentiation in mammalian cells. The standard 3i and LIF media include LIF to activate STAT3, CHIR99021 to inhibit GSK3, PD0325901 to inhibit MEK, and PD184352 to inhibit FGF receptor tyrosine kinase. We found that replacing PD184352 with A83-01, which inhibits TGF-beta, resulted in healthier looking proliferating colonies [13]. In our experiments, we used standard media conditions (except increased FBS to 15%, because we observed that the cells tend to proliferate faster in 15% FBS) and then added LIF plus the three inhibitors: PD0325901 (Stemgent), CHIR99021 (Stemgent), and A83-01 (Tocris Biosci) [12].

*cESC and 2i+ Media*. This medium is a combination of cESC and 2i+ (Table 1). We used standard media conditions, including 15% FBS levels, and then added LIF, 2i+ (PD0325901, CHIR99021, and A83-01), and the recombinant proteins (rhIL6, rhsIL6Ra, rmSCF, rhIGF1, and rhFGF2) at the same concentrations as above.

*Modified 2i+ Media for Avian Cells*. We tried various changes in the concentrations of LIF and the 2i+ molecules to derive a modified 2i medium for proliferation of avian cells. This medium was generated in the same manner as our 2i+ media (Table 1), but with reduced LIF (from 1000 U/ML to 50 U/mL), increased PD0325901 (from 0.5 *μ*M to 1 *μ*M), no change in CHIR99021 (3 *μ*M), and increased A83-01 (from 0.5 *μ*M to 1 *μ*M). This medium was used to grow our more proliferative iPSC-like avian cells that lasted past the 20th passage reported in [8] and still proliferating as of the time of this paper.

### 2.4. Generating and Maintaining Control Mouse iPSC

Our in house mouse iPSCs frozen stock cells were used for the control experiments. They were generated according to the protocol described in Rosselló et al. [8]. After the cells were thawed, they were cultured in parallel with chicken iPSC-like cells with cESC or modified 2i+ media.

### 2.5. Mitotic Inhibition of Fibroblast Feeders for Cell Culture

Mouse fibroblasts were expanded, trypsinized, irradiated, and stored in aliquots in liquid nitrogen. Irradiated cells were thawed and plated as needed one to five days before use. Fibroblasts extracted from chicken were plated overnight at ~70% confluency onto the desired cell culture plates for growing chicken iPSC-like cells. The cells were then mitotically inactivated with mitomycin-C (Sigma M-0503) at a concentration of 10 *μ*g/mL of media for 2.5 hours at 39°C. Afterwards, the cells were washed 3 times with 1xPBS (Gibco) and incubated overnight in the media needed for chicken iPSC-like growth. The chicken iPSC-like cells were trypsinized (Gibco, 25200-056) and then spun down and plated onto the mitotically inactivated mouse or chicken fibroblasts with fresh media.

### 2.6. Mechanical Extraction of iPSC-Like Colonies from Fibroblasts

Chicken iPSC-like cell cultures with a mix of stem cells and self-proliferating fibroblasts were incubated with 0.25% trypsin with EDTA (Gibco) for 30 seconds at room temperature, while gently rocking the plate back and forth. The trypsin was then neutralized with modified 2i+ media and the plate placed under a dissecting microscope. With a 500 *μ*L pipette, the stem cell colonies were then separated from the fibroblast and siphoned into the pipette tip. The extracted colony was then placed into a well on a cell culture plate at approximately 3–5 colonies per 22.1 mm or 6.35 mm well on a 12-well plate or 96-well plate, respectively, (Corning) with fresh modified 2i+ media. The extracted colonies were then cultured as other chicken iPSC-like cells at 39°C, 5% CO_2_, and 21% oxygen.

### 2.7. Alkaline Phosphatase Staining

Cells on petri dishes or glass slides were fixed with 4% paraformaldehyde in water for 10 minutes at room temperature. They were then washed 2-3 times with Tris maleate buffer (30 mM Tris pH 9.0) and stained for alkaline phosphatase activity for 30 minutes at room temperature with freshly prepared solution containing Naphtol-AS-MX-phosphate (Sigma N5000) and Fast Red TR Salt (Sigma F2768) in Tris maleate buffer with MgCl_2_ (10%). They were then washed with PBS to stop the color reaction and coverslipped with Vectashield HardSet mounting medium containing DAPI (Vector Labs, H-1500).

### 2.8. Neuronal Differentiation

We attempted to generate neurons from the chicken iPSC-like cells using two different protocols: (1) Stem Cell Technologies ES-Cult Kit, using the dish embryoid body approach exactly as described (Stem Cell Technologies, Cat#28706; production terminated); and (2) a differentiation method with modifications [15]. The modifications used 2 media supplements, N2 (Gibco 17502-048) and B27 (Gibco 17502-048), which are normally used in combination with LIF (leukemia inhibitory factor) to maintain the pluripotency of ESCs and progenitor cells. LIF is the main component of the culture condition that helps the cells retain their undifferentiated state. N2 and B27 without LIF are used to maintain neuronal cultures and to stimulate stem cells and progenitors to differentiate into neurons. For the method used by Lee et al. [15], there were three steps.

1. We first formed embryoid bodies (EBs) in 6 cm plates containing iPSC-like cells that were trypsinized with 0.25% trypsin plus EDTA (Gibco). The trypsin was quenched and the cells were spun down at 1500 rpm. The cells were then resuspended in* embryoid body formation media *(see below) and plated on ultralow adherent 6-well cell culture plates (Stem Cell Technologies, Cat#27145) at approximately 10^6^ cells per well for 4 days. Within 2 days, the cells clustered into EBs and when cultured for more than 4 days, in some cases, EBs started to show cardiac-like beats with a sporadic rhythm. Increasing cellular concentration per well increased the size and number of EBs.
2. After 4 days of EB formation, they were carefully transferred into 15 mL tubes and allowed to settle for 15–30 min via gravity to the bottom of the tube. The medium was carefully aspirated so as to not disturb the EBs. The EBs were then resuspended in* transition media* (see below) and plated onto poly-L-ornithine and laminin coated glass coverslips (BD BioCoat, 1232C71) in replicate in 24-well cell culture plates. After 2 to 3 days of culture the EBs attached completely to the coverslips.
3. We then continued to culture the cells in* N2B27 neuronal differentiation media* (see below) for 7–10 days to obtain functioning neurons. As needed, 2/3rd of the culture medium was replaced with fresh N2B27 media.

*Embryoid Body Formation Media*. This is standard culture medium without the cell proliferation inhibitors (Table 1). The specific concentrations of the standard ingredients were KO DMEM (81%), FBS (15%), sodium pyruvate (1 mM), nonessential amino acids (1 mM), Glutamax (1 mM), beta-mercaptoethanol (0.005 mM), and Penicillin/Streptomycin (1 mM).

*Transition Media*. This medium is a 1 : 1 mixture of our standard media (Table 1, from KO DMEM to Penicillin/Streptomycin) and N2B27 neural differentiation media based on Lee et al. [15], but without additional retinoic acid. N2B27 medium was made from a 1 : 1 mixture of N2 and B27 media, which are stored separately before use. N2 medium consists of DMEM/F12 (99%, Gibco), N2 (1%, Gibco 100x), and BSA (0.01%, Fisher); B27 consists of neurobasal media (96%, Gibco), B27 (2%, Gibco, 50x), Glutamax (1 mM), Penicillin/Streptomycin (1 mM), and beta-mercaptoethanol (0.005 mM). The final concentrations of all ingredients in the 1 : 1 mixed transition media of N2B27 and the standard media are KO DMEM (40.5%), FBS (7%), sodium pyruvate (1 mM), nonessential amino acids (1 mM), Glutamax (1 mM), beta-mercaptoethanol (0.005 mM), Penicillin/Streptomycin (1 mM), DMEM/F12 (24.5%), N2 (0.25%, Gibco 100x), neurobasal media (24%), and B27 (0.5%, Gibco 50x).

*N2B27 Neuronal Differentiation Media*. This medium is the same as the N2B27 described above, with final concentrations of DMEM/F12 (49%), N2 (0.5%), BSA (0.005%), neurobasal media (48%), B27 (1%, Gibco 50x), Glutamax (0.5 mM), Penicillin/Streptomycin (1 mM), and beta-mercaptoethanol (0.005 mM).

### 2.9. Immunocytochemistry

Differentiating neurons on glass coverslips were fixed with 4% paraformaldehyde in water for 15 minutes and washed with 1x PBS. The cells were then permeabilized with 0.3% triton X-100 in 1x PBS for 30 minutes at room temperature and blocked with appropriate serum or 1% BSA at room temperature for 1 hour. The cells were incubated with primary antibodies (NeuN, Millipore MAB377; TU-20, Millipore, MAB1637; or Nestin, Millipore, MAB353) for 1 hour at a 1 : 100 dilution in PBS and then a secondary antibody at a 1 : 1000 dilution for an hour as follows: NeuN was coupled with an AlexaFluor488 fluorescent secondary antibody (Molecular Probes); TU-20 and Nestin were coupled with an anti-mouse IgG secondary antibody (Sigma, A3562) that was then reacted with Fast Blue RR Salt (Sigma FBS25). The coverslips that the cells were grown on were then mounted on a glass slide with Vectashield mounting media containing DAPI. We quantified the number of NeuN labeled cells by counting all cells in a 100x view from three independent replicates, divided that number by the total DAPI labeled cells, and then averaged the values.

### 2.10. Electrophysiology

Coverslips with live neurons were transferred into the recording chamber containing standard extracellular solution (in mM: 140 NaCl, 3 KCl, 2 CaCl_2_, 1.25 MgCl_2_, 1.25 NaH_2_PO_4_, 20 D-glucose, and 10 HEPES). Whole-cell recordings were established using DIC optics on an upright microscope (Olympus BX51WI with LUMPLFLN40XW objective). Recording pipettes had resistances ranging from 3 to 6 MΩ and series resistances were <20 MΩ. Data were measured and acquired with an Axoclamp 700B amplifier and Digidata 1440 (Molecular Devices). Internal electrode solutions contained, in mM, 135 K gluconate, 2 MgCl_2_, 2 MgATP, 0.5 NaGTP, 0.5 EGTA, 10 HEPES, and 10 phosphocreatine (pH 7.35). Cells were driven by current injection to test for the presence of action potentials.

### 2.11. Quantitative Real-Time PCR

Cells were spun down and RNA was extracted using a standard kit (Promega SV total RNA isolation system, Z3105). Chicken iPSC-like cells were grown in our modified 2i+ media conditions, control fibroblasts were grown in the same media, and differentiated chicken neurons were grown in the differentiation media. RNA was quantified using a NanoDrop 2000c (Thermo Scientific, Waltham, MA) and stored in −80°C. Complementary DNA (cDNA) was produced (10 *μ*L of 2x RT buffer 1 *μ*L of 20x superscript II enzyme; Applied Biosystems) using the MJ tetrad system to run a reverse transcription cycle (cycle: 37°C for 60 min, 95°C for 5 min, 4°C hold). The cDNA was then used as a template to perform gene expression assays using the BioRad Cx96 real-time machine with either custom TaqMan primer probes (generated by Applied Biosystems) or custom primers with 1 *μ*L SYBR green (BioRad), in 20 *μ*L reactions containing 1 *μ*L template (~2 *μ*g/*μ*L), 10 *μ*L 2x Gene Expression Master Mix (BioRad, Hercules, CA). To discriminate between relative expressions of neuronal and stem cell genes, different primers were generated for mouse and chicken species, using nonoverlapping sequences. The probes included chicken specific Nanog (Gene ID NM_001146142.1, 5′-CAGCAGACCTCTCCTTGACC-3′, rev primer 3′-TTCCTTGTCCCACTCTCACC-5′), Oct4 (Gene ID NM_001110178, 5′-GGGAAGATGTTCAGCCAGAC-3′, rev primer 3′-GTCTGGTTCTGCAACCGGCG-5′), and Sox2 (Gene ID NM_205188.1, 5′-TTTCCTAGGGAGGGGTATGAA-3′, rev primer 3′-GCAGAGAAAAGGGAAAAAGGA-5′) to determine the stemness of the chicken iPSC-like cells; DCX (double cortin, Gene ID NM_204335, 5′-CGCTGAAAACCGCTTCAGAT-3′, rev primer 3′-ACTGCTCGAGGTCCCATTTG-5′), Nestin (Gene ID NM_205033.1, 5′-GCAGAGCCAGAGCGCACCAA-3′, rev primer 3′-CAGGCTCAGCCCCACTGTGC-5′), and beta-tubulin III (Gene ID NM_001031598, 5′-GGCCTCCTCTCACAAGTACG-3′, rev primer 3′-AAATGAAGTTGTCGGGGCGG-5′) for neural specificity; and GMFB (Glia Maturation Factor B, Gene ID XM_418091.4, 5′-CGGGCAGGATGGATTTCTCT-3′, rev primer 3′-GTACTCATTGGCCTCGTGCT-5′) for glial specificity. All primers were tested and showed efficiency levels above 90%, consistent with the MIQE guidelines [16]. Each well contained 10x RT buffer (200 mM Tris-HCl/pH 8.4; 500 mM KCl), 25 mM MgCl_2_, 1 *μ*L of cDNA product, 10 mM dNTP, and iTaq DNA polymerase. Cycling conditions for the RT-PCR were as follows: 48°C for 10 minutes and 95°C for ten minutes, followed by 40 cycles of 95°C for 15 seconds (Denaturation) and 60°C for 1 minute (annealing). No-template control analyses were run for each primer set, probe, and telomerase assay. 18S rRNA endogenous control was run for each sample (TAQMAN MAN PRIMER: Cat# Eukaryotic 18S rRNA HS99999901_S1; Applied Biosystems). All reactions were performed in quintuplets. The results were normalized to the endogenous 18S rRNA expression (ABI) and to the gene expression level of control groups using the DDCT method [17]. An analysis of variance was performed for variations in gene expression within each species. Statistical significance was measured at *P* = 0.05.

### 2.12. MTT Assay

The MTT assay was performed with a standard kit (Promega SV Cell Titer 96 nonradioactive cell proliferation assay, G4000). Chicken fibroblasts and chicken iPSC-like cells were grown in modified 2i+ media. After each passage, the cells were incubated for 24 hours, and the kit dye solution was added to each well and incubated per kit protocol at 37°C for 4 hours. Afterwards, the solubilization buffer was added to each well per protocol and incubated overnight, and the absorbance was read at 570 nm.

## 3. Results

### 3.1. Maintenance of Chicken iPSC-Like Cells

The purpose of the first part of our study was to find conditions that would allow us to grow avian iPSC-like cells past the 5th passage, which we had difficulty doing in cESC media [8]. Different media conditions were tried with a variety of cells, including both chicken embryonic stem cells obtained from Bertrand Pain, chicken primordial germ cells from Marie-Cecile van de Lavoir, and chicken iPSC that we derived ourselves. Here we report on five media conditions for comparative purposes, using the previous generated iPSC-like cells grown in cESC media including the previous media conditions as a benchmark. For our general protocol, chicken embryonic fibroblast cells were transfected with the STEMCCA cassette containing the four inducing mouse transcription factors, and nontransfected chicken embryonic fibroblasts were used as controls, in standard media conditions in replicates of 12–24 wells. After 1 week, the cells were passaged once and then transferred and maintained initially in one of four differentiation inhibiting media conditions in replicates of 4: BRL-conditioned Plus, cESC, 2i+, cESC, and 2i+ (Table 1: see Section 2 for detailed media compositions). Previous findings have shown and our own results have validated (not shown) that BRL-conditioned [18] and cESC media [11] were sufficient for maintaining chicken primordial germ cells (PGCs) and chicken ESCs, respectively, and that 2i+ medium was sufficient for maintaining mouse stem cells* in vitro* [12].

**Figure 1:**
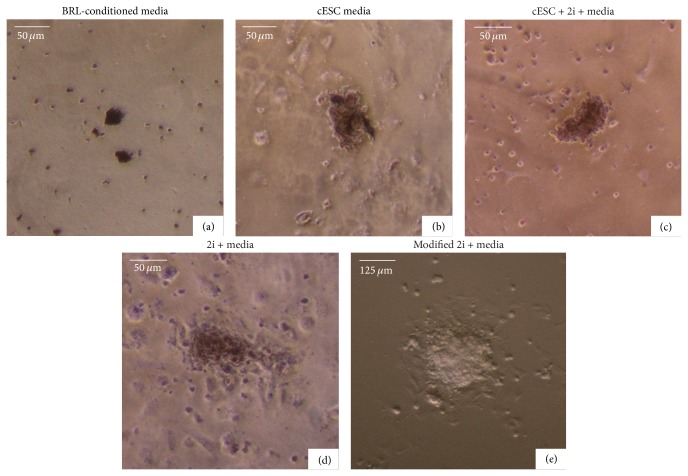
Examples of chicken iPSC-like cells grown in different media conditions during 2nd passage growth. (a) Chicken iPSC-like cells grown in BRL-conditioned media. The cells became darker and detached from the bottom of the plate. They did not survive past the 2nd passage. (b) Cells grown in cESC media initially proliferated well but began to detach from the plate after the 2nd passage. They did not survive past the 4th passage. (c) Chicken iPSC-like cells grown in cESC and 2i+ media did not survive past the 4th passage. (d) Chicken iPSC-like cells grown in 2i+ media did not survive past the 6th passage. (e) Chicken iPSC-like cells grown in modified 2i+ media. The colony was much bigger than the colonies grown in the other 3 media conditions, and the scale bar is adjusted likewise.

In our experiments, in all media conditions the chicken cells began to form small iPSC-like colonies of proliferating cells within the 1st-2nd passages (Figure 1), whereas the fibroblasts did not. However, between the 2nd and 5th passages there were differences between conditions (quantifications in Table 2). The colonies in the BRL-conditioned media were very small and dark and looked poor, and all of them quickly senesced by the 2nd passage (within several weeks). Senescence was characterized by seeing a few to no remaining colonies or proliferative cells. The cells in the cESC and 2i+ media lasted until the 4th passage, but in only ~50–70% of replicates, and then all of them senesced by the 5th passage (Table 2). The cells did not grow better in cESC + 2i+, in that only about 50% of the cells made it to passage 6 and then stopped growing (Table 2). We then generated a number of other modifications of the 2i+ media (2i+ Mod) with LIF by systematically lowering and increasing inhibitors (0.5 *μ*M increments) in the media. We tested the different formulations of the media on chicken iPSC-like cells and we could clearly identify which media aided the cells' growth by the 3rd or 4th passage. We found that lowering LIF and increasing two inhibitors (PD0325901 and A83-01) resulted in chicken colonies that appeared healthier and larger already by the 2nd passage compared with the 1st passage (Figure 1(e)) and grew well past 20 passages (up to 8th shown in Table 2) and at a similar rate as our mouse iPSC controls (Table 2). Our mouse iPSCs grew well with proliferating colonies in both cESC and the modified 2i+ media (Table 2), indicating that the differences seen with chicken using cESC media could reflect species differences.

**Figure 2:**
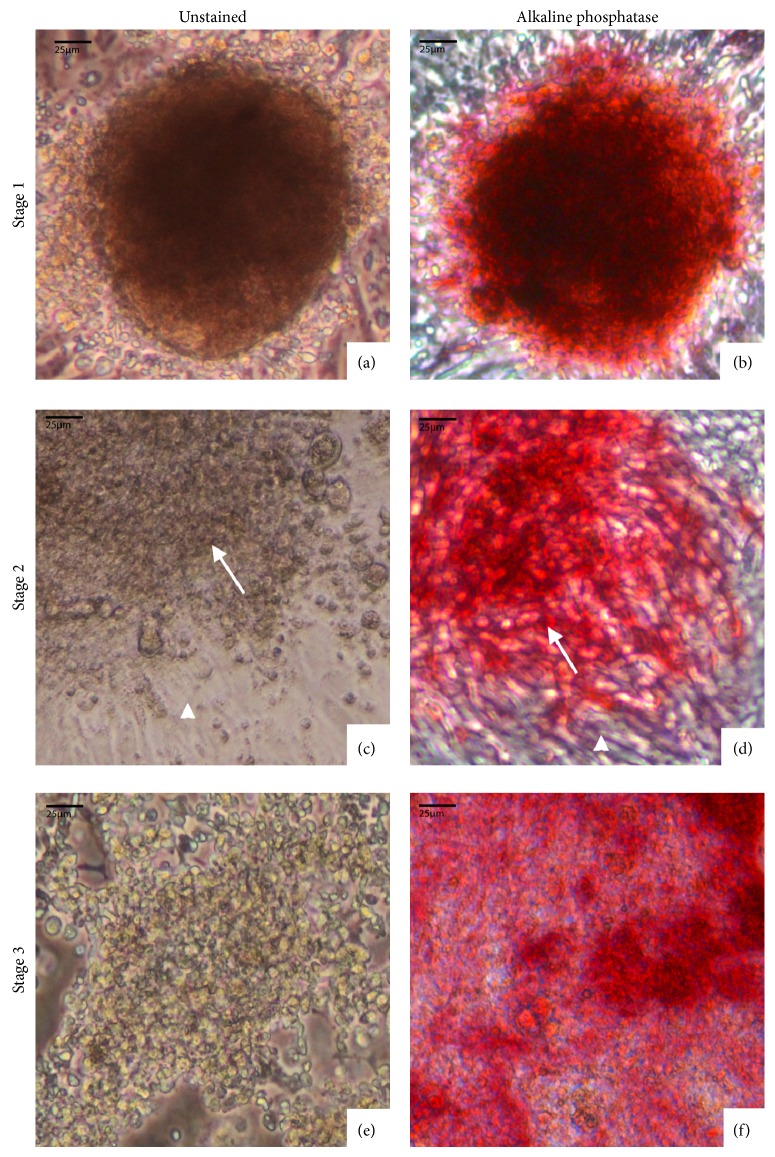
Three stages of chicken iPSC-like cells morphological development in modified 2i+ media. (a) Stage 1: chicken iPSC-like cell colony during the 4th passage with a characteristic dark center. (b) Comparable colony as in (a) stained with alkaline phosphatase. (c) Stage 2: colony is lighter by the 8th passage with fibroblasts radiating from the stem cell colony. (d) Comparable colony as in (c) stained with alkaline phosphatase; the differentiated fibroblasts do not stain with alkaline phosphatase. Arrow, colony; arrowhead, fibroblast. (e) Stage 3: 10th passage, larger colony, and fewer fibroblasts underneath or on the sides. (f) Comparable colony as in (e) stained with alkaline phosphatase.

Unlike the other media conditions, we found that the morphology of the chicken iPSC-like colonies in the modified 2i+ media repeatedly developed through three distinct stages. When the cells were first transduced, the colonies exhibited a clearly rounded shape with a darkly pigmented center (Figure 2(a)), that is, representative of the morphology of chicken embryonic stem cells [8]. After approximately 4-5 passages, when the colonies in the other media conditions began to senesce, the colonies in the modified 2i+ medium lost their pigmentation and fibroblasts began to appear around the periphery of the colony (Figure 2(c)). The colonies in each subsequent passage would consistently start growing at a steady rate (requiring a 1 : 2 dilution every 2-3 weeks) first without the fibroblasts, which then would appear several days later. When we removed the fibroblasts in the periphery mechanically with a 500 *μ*L pipette (*n* = 5), the cells at this stage stopped proliferating. When we plated them on mitomycin-C-treated or irradiated mouse or chicken feeder fibroblasts (*n* = 3 each), stem-like cell proliferation even more dramatically decreased. Thus, the only way we were able to maintain growth was to let the cells in the modified 2i+ media generate the peripheral fibroblasts during growth at this stage. However, after the 8-9th passage, the majority of colonies (>65% ± 12%) began to lose development of fibroblasts and their rounded morphology (Figure 2(e)), although, even as in our regular mouse iPSCs and ESCs, there was still a small amount of differentiated fibroblast-like cells in the culture wells. Finally, after about the 12th passage, the cell colonies began to resemble the morphology of mouse iPSCs and proliferated at a similar rate (1 : 4 dilution every ~7 days) and as reported in the literature for mice [3]. We thus far have been able to maintain this passage rate for the chicken iPSC-like cells for more than a year, as opposed to a 1 : 2 dilution every 2-3 weeks for the previous stages. All the colonies throughout the three stages still stained positive for alkaline phosphatase (Figures 2(b), 2(d), and 2(f)) and expressed endogenous chicken homologs of the stem cell inducing genes (also see Rosselló et al. [8]); the surrounding fibroblasts did not stain for alkaline phosphatase. Thus, our modified 2i+ media for avian cells consistently achieved our goal of creating the conditions for continued growth and development of avian iPSC-like cells.

**Figure 3:**
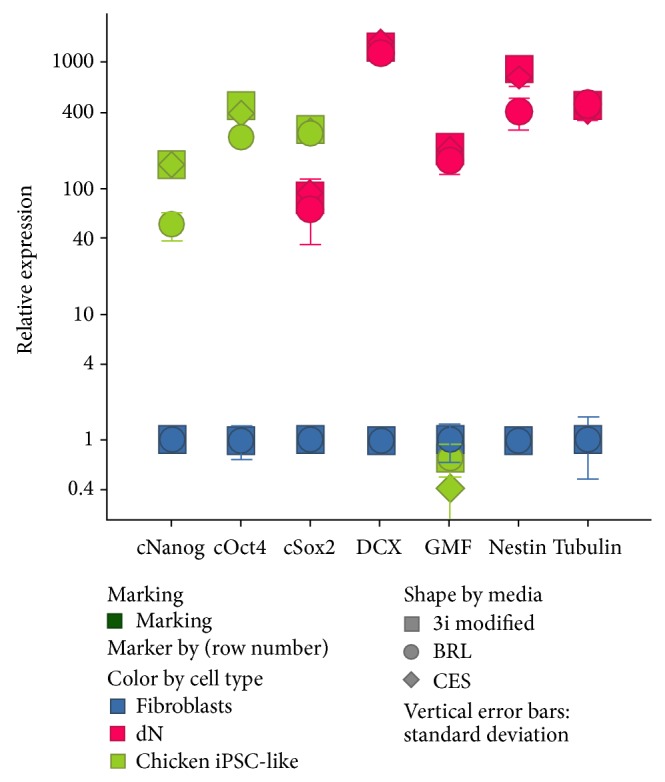
qRT-PCR of chicken iPSC-like cells at the 7th passage and differentiated neurons in comparison to chicken embryonic fibroblasts. cNanog, cOct4, and cSox2 are chicken homologs of genes normally expressed in stem cells relative to fibroblast; chicken Sox2, DCX, Nestin, and beta-tubulin III are in neurons and GMFB in glial cells. The differentiated neuronal culture used was from 10 days after induction. Values below 0.4 are not shown in the figure (barely detectable). DCX and tubulin had no (zero) detectable expression in chicken iPSC-like cells cultured in 2i+ modified media. cNanog and cOct4 had no detectable expression in differentiated neurons. Glia maturation factor had 0.72 ± 0.21 relative expressions and Nestin had 0.0047 ± 0.00034 in PRPSCs. The values are from *n* = 3 independent replicates. Error bars, SEM. *P* < 0.05, relative to fibroblast controls.

**Figure 4:**
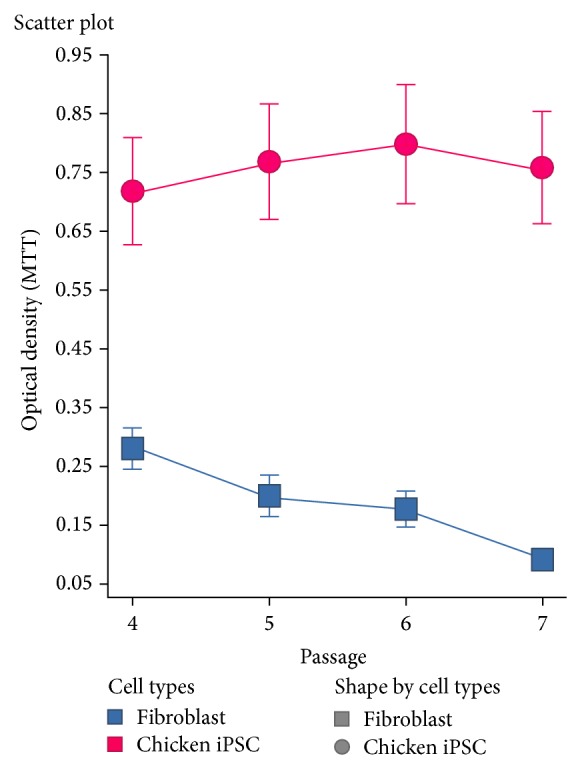
MTT assay of chicken iPSC-like cells compared to chicken embryonic fibroblasts. Fibroblasts decreased significantly in optical density as they were passaged over time, while chicken iPSC-like cells grown in modified 2i+ retained their metabolic potential regardless of passage number.

Colonies throughout the three stages also expressed endogenous chicken homologs of the stem cell inducing genes (Figure 3; also see Rosselló et al. [8]) and had a normal karyotype by the last stage (shown in Rosselló et al. [8]). Here we examined the highly proliferative colonies. Using qRT-PCR with 3 chicken specific probes known to associate with pluripotency, we found that relative to nontransduced fibroblasts, the highly proliferative chicken iPSC-like cells still expressed high levels of endogenous Nanog, Oct4, and Sox2 (Figure 3); endogenous klf-4 was low, as in the earlier stages in normal chicken ESC (Figure 3; [8]). Because the chicken iPSC-like cells were transduced with mouse homologues of Oct4, Sox2, c-myc, and klf-4, the significant increased expression of the chicken homologues, including Nanog, a nontransduced gene, indicates that the endogenous pluripotent machinery was still active throughout the last stage. Similarly, MTT assays indicated that chicken iPSC-like cells retained a significantly high proliferation (metabolic) ability (Figure 4).

### 3.2. Neuronal Differentiation of Chicken iPSC-Like Cells

We took the cells that made it past the 5th, 8th, 12th, and 20th passages in modified 2i+ media and tried to differentiate them into neurons. We first tried a protocol developed by Stem Cell Technologies, ES-Cult (Stem Cell Technologies, Cat#29706; terminated), but found that although they were able to generate neuronal-like appearing cells for mouse, the density of differentiated mouse cells was sparse and the avian embryoid bodies (the first step) had trouble attaching to the plates. We then tried and modified the method of Lee et al. [15], without additional retinoic acid, and used our avian standard media composition. We found that, in this modified neuronal differentiation medium, the chicken iPSC-like cells went through the five distinct stages of differentiation.

**Figure 5:**
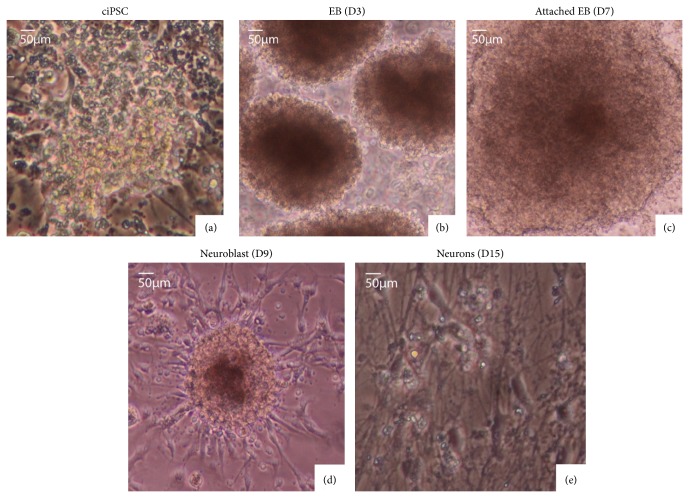
Differentiation of chicken iPSC-like cells (ciPSC), grown in modified 2i+ media, into neurons. (a) Undifferentiated chicken iPSC-like cells in modified 2i+ media at passage 10. (b) Embryoid bodies in suspension culture in embryoid body media, 2 days after initiation of differentiation protocol. (c) Attached embryoid body in transition media, 6 days after initiation of differentiation. (d) Neurite outgrowth (arrow) in N2B27 media at day 9 since the start of differentiation. (e) Developed neurons (arrows) in N2B27 media at day 15.

In the first stage, when the chicken iPSC-like cells (Figure 5(a)) were placed in embryoid body formation medium and on ultralow adherent plates, they detached from the bottom of the plate, became pigmented, and formed a 3-dimensional ball, that is, EB (Figure 5(b)). When these EBs were then placed into neuronal transitional media on glass coverslips with poly-L-ornithine and laminin for several days, they attached to the coverslips and began to flatten and spread out (Figure 5(c)). When they were then placed in N2B27 media, during the next 7 days, the cells migrated away from the EB and began to form neurite processes (Figure 5(d)). After 12 to 14 days in N2B27 media, these differentiating cells showed clear neuronal morphology (Figure 5(e)). The cells remaining in the original EBs themselves took on a neurobasal-like morphology, which have rosette-like structures (Figure 5(d); [19]).

**Figure 6:**
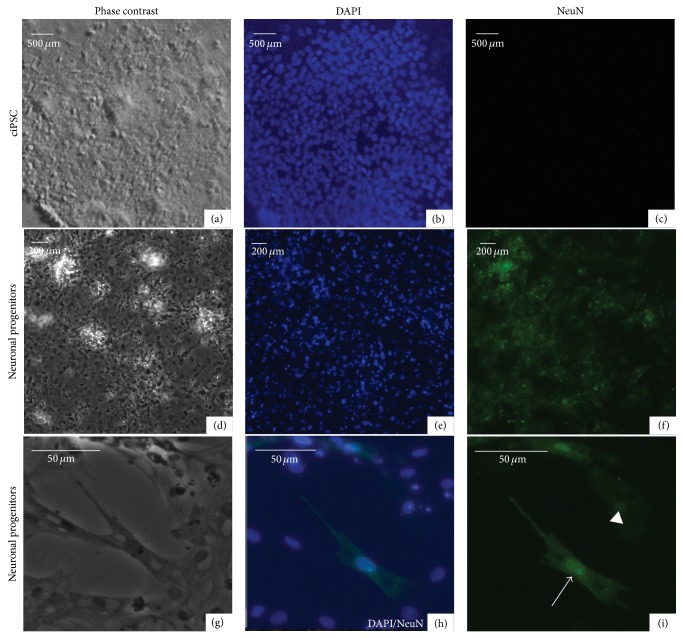
Neuronal marker staining of differentiating neurons. ((a)–(c)) Undifferentiated chicken iPSC-like cells under phase contrast (a) or with fluorescence for DAPI (b) and NeuN (c) stains. ((d)–(f)) Neuronal differentiated chicken iPSC-like cells under phase contrast (d) or with fluorescence for DAPI (e) and NeuN (f) stains at day 7. ((g)-(h)) A close-up of single cells labeled for NeuN and DAPI. In the close-up, we chose a region with fewer NeuN labeled cells in order to show the contrast between labeled and unlabeled cells. Arrow, example of strongly labeled NeuN cells; arrowhead, example of moderately labeled NeuN cells. The control iPSC-like cells had nearly no labeled cells (only 0–2 per entire well or plate).

We found that differentiating cells already by day 7 expressed NeuN (Figure 6(i)), a neuronal nucleus antigen [20]. This was even the case for some of the cells that did not yet have clear neuronal processes, suggesting that the cells are neuron precursors. The NeuN labeled cells tended to be located further away from the neurobasal cells (Figure 6(f)). The colocalization of the NeuN staining (green) and DAPI nucleus stain (blue) indicates that the antigen was correctly expressed mainly in the nucleus of the cell and to a lesser extent in the cytoplasm (Figures 6(h) and 6(i); [20]). At this stage, the density of labeled NeuN cells was 56% (±11.6%) for cells differentiated from the 5th or 8th passage and 56.3% (±5.46%) for cells differentiated from the 12th and 20th passages. In contrast, wells with only the starting chicken iPSC-like cells at a similar density had between 0 and 2 NeuN labeled cells in the entire well.

**Figure 7:**
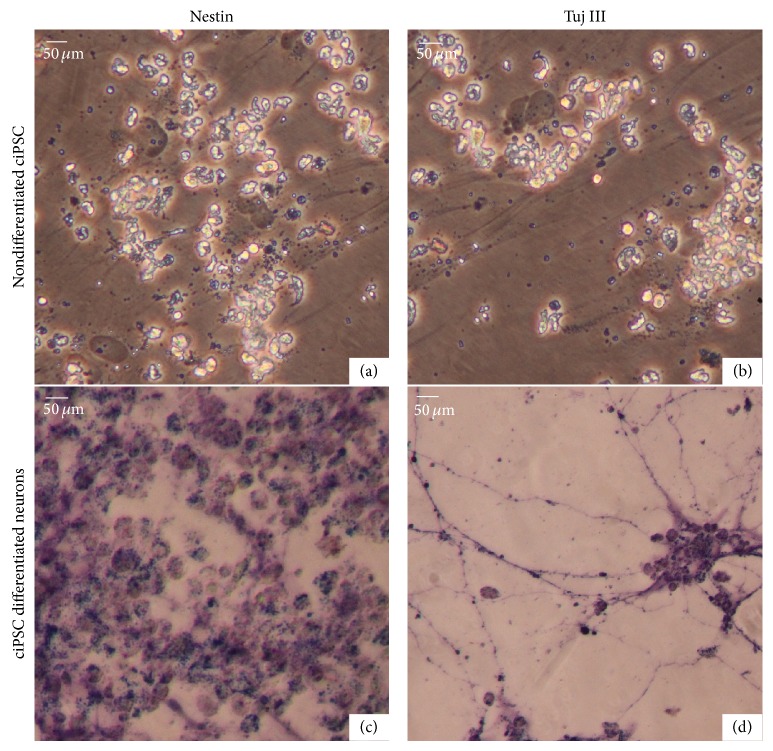
Neural marker staining of more fully differentiated neurons. (a) and (b) Nestin and beta-tubulin III (TU-20) labeling of nondifferentiated chicken iPSC-like cells. iPSC-like cells have shiny appearance under the light settings used. (c) and (d) Differentiated neurons from chicken iPSC-like cells stained positive for Nestin and beta-tubulin III at day 14 (purple label).

After 14 days of neuronal differentiation, the cells stained for Nestin, intermediate filament protein, that is, preferentially expressed in the cytoplasm of neuronal soma (Figure 7(c)) [21], and stained for beta-tubulin III (TU-20), which labels neuronal microtubules that are often expressed in neuronal axons and dendrites (Figure 7(d)). The TU-20 label allowed us to visualize the neuronal processes, where it could be seen that cells formed connections or at least contacted each other through these processes across long distances on the coverslip (Figure 7(d)). Such long processes were only seen in the older cultures from around 14 days onward. The starting chicken iPSC-like cells did not show differentiated processes and did not stain for NeuN, Nestin, or TU-20 (Figure 6(c) and Figures 7(a) and 7(b)). These results demonstrate that the differentiated cells exhibit neuronal morphology and antigens not present in the starting cells.

The neuronal identity of the differentiated cells was further supported by qRT-PCR of the neurons with the neuronal markers doublecortin, Nestin, and beta-tubulin III in the chicken iPSC-like cells (Figure 3) at significantly higher relative expression to the iPSC and fibroblast or iPSC-like controls. We also found significantly greater expression of glial marker glial maturation factor beta (GMFB) in the differentiated cells than in the fibroblasts and ciPSC-like cells. This suggests that, in addition to neurons, some of the chicken iPSC-like cells may be differentiated into glial cells in our neuronal differentiation media.

**Figure 8:**
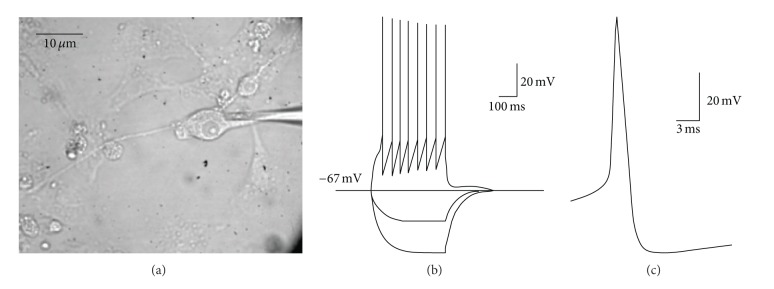
Whole-cell current-clamp recordings of differentiated chicken neurons generated from iPSC-like cells reveal action potential firing. (a) DIC image showing a single cell (arrow) with recording pipette in whole-cell configuration (arrowhead). (b) Membrane potential responses generated by current injection. Suprathreshold depolarizing currents resulted in trains of overshooting action potentials. (c) Expanded view of a single action potential illustrating narrow spike width and strength after hyperpolarization.

We next tested whether the differentiated cells exhibit membrane excitability characteristic of neurons. We made whole-cell recordings from cultured cells under visual control, selecting cells with a multipolar neuron-like morphology (Figure 8(a)). Of the 4 cells that we patch-clamped, 3 exhibited strong action potentials. Consistent with a neuronal phenotype, the differentiated cells had hyperpolarized resting membrane potentials between −68 and −65 mV. We then injected depolarizing currents in current-clamp mode to evaluate the presence of action potential firing (Figure 8(b)). This consistently generated trains of overshooting action potentials that were evoked at a threshold of approximately −40 to −33 mV and had heights from 78 to 86 mV. Spikes were rapid (width at half max, 1.5 msec) and also showed prominent after-hyperpolarizations (Figures 8(b) and 8(c)). Hyperpolarizing current injections produced graded changes in membrane potential indicating an input resistance of 433 megaohms. Each of the multipolar cells that we recorded from showed similar firing properties. Thus, differentiated iPSC-like cells displayed several electrophysiological features indicative of a neuronal cell type.

## 4. Discussion

Our study demonstrates an efficient way to generate and maintain chicken iPSC-like cells and to differentiate them into neurons. The findings suggest that the prior difficulty in getting the cells to proliferate past a small number of passages was not necessarily due to whether the cells were iPSCs or not, but due to media conditions specific for individual species. However, our results also indicate that the media conditions can help transition cells from a potentially partial to a more highly proliferative reprogrammed state. Our chicken iPSC-like cells maintained in modified 2i+ avian media developed through three different morphological stages as the cells were passaged: slow growth, more rapid growth with differentiating fibroblasts, to rapid growth with little fibroblasts. We hypothesize that the surrounding fibroblasts differentiate from the iPSC-like cells and help sustain the proliferating stem cells in the early stages, as demonstrated for some human [22] and mouse ESC lines [23]. This is most likely the reason why protocols for maintaining human stem cells benefit from coculturing the cells with fibroblasts [22]. In such cases, it is thought that the stem cells, through asymmetric divisions, create their own niche of differentiating feeder cells that allow further proliferation of the stem cells. The lack of growth of our chicken cells on the mouse and chicken feeder cells indicates that self-derived chicken fibroblasts might be producing factors specific to supporting the chicken stem cells. Overall, the different stages indicate that, under the right conditions, iPSC development can be a gradual process. We do not know if species differences between mouse and chicken reflect differences between mammals and birds, but some preliminary results suggest that this might be the case at least partially; ongoing experiments maintaining iPSC-like cells which we generated from zebra finches (a songbird) show that the finch cells in our modified 2i+ are more proliferative than in any of the other media (unpublished observations).

One of the main findings that we encountered in this paper is increased ability for the cells to proliferate when we decreased the amount of LIF in the media condition by nearly 20-fold. LIF is considered an antidifferentiation factor that is needed for stem cells to maintain their pluripotency and removal of LIF initiates stem cell differentiation [24, 25]. We have found that when we decrease the amount of LIF in the culture media, some of the chicken iPSC-like cells will differentiate and form a niche on which more stem cells will further proliferate. We believe that this is the reason for the transformation seen in the chicken iPSC-like cells to become fully immortalized. Similarly, we deviated away from the standard 3i media by replacing PD183452, which inhibits FGF receptor tyrosine kinase, with A83-01, which inhibits TGF-beta, and found that this was an agreeable replacement for culturing chicken and mouse iPSC-like cells [13]. By replacing PD184352 with the more potent MEK inhibitor PD0325901, Ying et al. found that the “2i” combination of CHIR99021 and PD184352 was highly efficient in maintaining pluripotent mouse ES cells. Ding and colleagues introduced A83-01, a selective inhibitor of TGF-betaRI, ALK4, and ALK7, to generate rat and human iPSCs with a self-renewal state closer to mouse ES cells [26]. Different from mouse ES and iPS cells, growing rat and human iPSCs in 2i medium still resulted in differentiation, which was successfully prevented by the addition of A83-01. This implies that unlike mouse epiblast stem cells and more like rat and human iPSCs, chicken iPSC-like cells require activation, not inhibition of TGF-beta/activin pathways.

From the chicken iPSC-like cells, we were able to repeatedly generate action potential producing neurons in a 3-stage process, which in the last stage took 14 days to become mature and functionally active. Another study conducted in parallel with ours [27] was able to differentiate quail iPSC-like cells into cells with neuron structure but did not test if these cells were functional. The 14 days are equivalent to primary embryonic neurons from late gestation day rats or mice and the time it takes (between 10 and 14 days)* in vitro* for mouse stem cells to become neurons capable of firing action potentials [15]. Thus, this last stage would be comparable to neuron maturation after birth; the previous stages would be neuronal precursor development during gestation.

## 5. Conclusions

This study contributes to a progressive series of experiments showing that it is possible to create nonmammalian iPSC-like cells and control their differentiation* in vitro*. The differentiation of avian iPSC-like cells into neurons opens up possibilities to study fate and function of neurons from one avian species transplanted into another species.

## Figures and Tables

**Table 1:** Ingredients of the five passaging media conditions presented in this study.

Media name	BRL-C. plus	cESC	2i+	cESC 2i+	2i+ mod.
KO DMEM	82%	87%	82%	87%	82%
FBS	15%	10%	15%	10%	15%
Na Pyruvate (100 mM)	1 mM	1 mM	1 mM	1 mM	1 mM
Glutamax (100x)	2 mM	1 mM	1 mM	1 mM	1 mM
Nonessential amino acids (100x)	1 mM	1 mM	1 mM	1 mM	1 mM
Beta-mercaptoethanol	0.005 mM	0.005 mM	0.005 mM	0.005 mM	0.005 mM
Penicillin/Streptomycin (100x)	1 mM	1 mM	1 mM	1 mM	1 mM
Nucleoside (100x)	1 mM	—	—	—	—
LIF	—	1000 U/mL	1000 U/mL	1000 U/mL	50 U/mL
PD0325901	—	—	0.5 *μ*M	0.5 *μ*M	1 *μ*M
CHIR99021	—	—	3 *μ*M	3 *μ*M	3 *μ*M
A83-01	—	—	0.5 *μ*M	0.5 *μ*M	1 *μ*M
rhIL6	—	1 ng/mL	—	1 ng/mL	—
rhsILR6a	—	1 ng/mL	—	1 ng/mL	—
rmSCF	—	1 ng/mL	—	1 ng/mL	—
rhIGF1	—	1 ng/mL	—	1 ng/mL	—
rhFGF2	—	1 ng/mL	—	1 ng/mL	—

The media are all similar in a set of standard cell culture media ingredients (from KO DMEM to Penicillin/Streptomycin), but differ in the presence and concentrations of some of those ingredients, as well as LIF, 2i+ inhibitors (PDO325901, CHIR99021, and A83-01), and recombinant human or mouse cytokine factors (rh or rm) that inhibit cell differentiation.

**Table 2:** Induced pluripotent stem cell rate of passage survival in different media conditions.

Passage	Chicken					Mouse	
	BRL-C. plus	cESC	2i+	cESC 2i+	2i+ mod.	cESC	2i+ mod.
1	2/4	4/4	4/4	4/4	2/2	4/4	1/1
2	0/2	4/4	3/4	4/4	8/8	4/4	4/4
3	—	3/4	2/2	3/4	4/4	4/4	4/4
4	—	4/4	2/2	2/2	4/4	4/4	4/4
5	—	0/4	0/2	1/2	3/4	4/4	4/4
6	—	—	—	0/2	4/4	4/4	4/4
7	—	—	—	—	4/4	4/4	4/4
8	—	—	—	—	4/4	4/4	4/4

Each row indicates a passage from the previous row. The numerator of the fraction indicates the number of cultures (wells/plates) that survived the passage, and the denominator represents the number of cultures started in that passage. A “—” sign indicates that no cells survived to make it to that passage. During each passage, if the cultures were highly proliferative, each well or plate of cells was expanded into 4 other wells/plates; if they had a low level of proliferation, they were only expanded to 2 others; if they were not proliferating, they were necessarily passaged undiluted from one well to another until the cells senesced. The denominator of the cell immediately inferior indicates the ratio at which the cells were split. We tried increasing or decreasing cell concentration for those cultures that stopped proliferating, but this did not prevent them from senescing. The chicken iPSC-like cells in our modified 2i+ media (2i+ mod.) and our own mouse iPSC in KO-DMEM media have both made it past 20 passages (not shown).
